# Hemorrhagic and thrombotic manifestations in the central nervous system in COVID-19: A large observational study in the Brazilian Amazon with a complete autopsy series

**DOI:** 10.1371/journal.pone.0255950

**Published:** 2021-09-10

**Authors:** Monique Freire Santana, Carlos Henrique Michiles Frank, Taynná Vernalha Rocha Almeida, Christiane Maria Prado Jeronimo, Rebecca Augusta de Araújo Pinto, Yasmin Ferreira Martins, Maria Eduarda Leão de Farias, Bruna Guimarães Dutra, José Diego Brito-Sousa, Djane Clarys Baía-da-Silva, Mariana Simão Xavier, Marcus Vinicius Guimarães Lacerda, Fernando Fonseca Almeida Val, Gisely Cardoso Monteiro, Vanderson de Souza Sampaio, Wuelton Marcelo Monteiro, Luiz Carlos de Lima Ferreira

**Affiliations:** 1 Departamento de Ensino e Pesquisa, Fundação Centro de Controle de Oncologia do Estado do Amazonas–FCECON, Manaus, AM, Brazil; 2 Universidade do Estado do Amazonas, Programa de Pós-Graduação em Medicina Tropical, Manaus, AM, Brazil; 3 Fundação de Medicina Tropical Doutor Heitor Vieira Dourado, Manaus, AM, Brazil; 4 Programa de Pós-graduação em Ciências da Saúde, Faculdade de Medicina, Universidade Federal do Amazonas, Manaus, AM, Brazil; 5 Departamento de Patologia e Medicina Legal, Hospital Universitário Getúlio Vargas, Universidade Federal do Amazonas, Manaus, AM, Brazil; 6 Instituto de Pesquisas Leônidas & Maria Deane, Fundação Oswaldo Cruz, Manaus, AM, Brazil; 7 Instituto Nacional de Infectologia Evandro Chagas, Fundação Oswaldo Cruz, Rio de Janeiro, RJ, Brazil; 8 Fundação de Vigilância em Saúde do Amazonas, Manaus, AM, Brazil; University of South Carolina, UNITED STATES

## Abstract

SARS-CoV-2 affects mainly the lungs, however, other manifestations, including neurological manifestations, have also been described during the disease. Some of the neurological findings have involved intracerebral or subarachnoid hemorrhage, strokes, and other thrombotic/hemorrhagic conditions. Nevertheless, the gross pathology of hemorrhagic lesions in the central nervous system has not been previously described in Brazilian autopsy cases. This study aimed to describe gross and microscopic central nervous system (CNS) pathology findings from the autopsies and correlate them with the clinical and laboratory characteristics of forty-five patients with COVID-19 from Manaus, Amazonas, Brazil. Forty-four patients were autopsied of which thirty-eight of these (86.36%) were positive by RT-PCR for COVID-19, and six (13.3%) were positive by the serological rapid test. Clinical and radiological findings were compatible with the infection. The patients were classified in two groups: presence (those who had hemorrhagic and/or thrombotic manifestations in the CNS) and absence (those who did not present hemorrhagic and/or thrombotic manifestations in the CNS). For risk assessment, relative risk and respective confidence intervals were estimated. Macroscopic or microscopic hemorrhages were found in twenty-three cases (52,27%). The postmortem gross examination of the brain revealed a broad spectrum of hemorrhages, from spots to large and confluent areas and, under microscopy, we observed mainly perivascular discharge. The association analyses showed that the use of corticosteroid, anticoagulant and antibiotic had no statistical significance with a risk of nervous system hemorrhagic manifestations. However, it is possible to infer a statistical tendency that indicates that individuals with diabetes had a higher risk for the same outcome (RR = 1.320, 95% CI = 0.7375 to 2.416, p = 0.3743), which was not observed in relation to other comorbidities. It is unknown whether the new variants of the virus can cause different clinical manifestations, such as those observed or indeed others. As a result, more studies are necessary to define clinical and radiologic monitoring protocols and strategic interventions for patients at risk of adverse and fatal events, such as the extensive hemorrhaging described here. It is imperative that clinicians must be aware of comorbidities and the drugs used to treat patients with COVID-19 to prevent CNS hemorrhagic and thrombotic events.

## Introduction

The coronavirus disease (COVID-19) is caused by a new type of beta coronavirus that is a positive-sense single-stranded RNA virus, and belongs to the *Coronaviridae* family. This virus has a genome similar to the viruses of the severe acute respiratory syndrome (SARS-CoV-1) and the Middle East respiratory syndrome (MERS-CoV), both categorized as severe respiratory syndromes [1,2]. The infection was first reported in December 2019 in the city of Wuhan, Hubei province, China, and was officially named COVID-19 on February 11^th^, 2020, by the World Health Organization (WHO) [3].

The disease affects mainly the lungs, however, different types of neurological manifestations, including headache, paresthesia, and impaired consciousness have been described during the disease [4,5]. The anosmia and/or ageusia reported in 88% of patients suggest neuroinvasion by the virus in the central nervous system (CNS) through the olfactory route [6]. In the first published cohort of cases with neurological manifestations, of 214 patients examined, 78 (36.4%) had neurologic manifestations. The authors reported that patients with severe infection were more likely to develop acute cerebrovascular disease, impaired consciousness, and skeletal muscle injury [7]. Other published cases also reported meningitis/encephalitis, stroke, cerebral venous sinus thrombosis, and acute hemorrhagic necrotizing encephalopathy [8–11].

SARS-CoV-2 (severe acute respiratory syndrome-related coronavirus 2) infections induce the systemic inflammatory response and may cause an imbalance between procoagulant and anticoagulant homeostatic mechanisms. According to a study of 388 patients from Italy, which describes the rates and characteristics of venous and arterial thromboembolic complications, such events occurred in 21% of the patients and included venous thromboembolism, ischemic stroke, and acute coronary syndrome [12]. In addition, high levels of D-dimer and severe platelet reduction have been reported as indicators of acute cerebrovascular events in patients with severe infection [13]. However, the mechanism that causes the hypercoagulable or hemorrhagic state and the frequency of fatal complications related to this event are unknown. In fatal cases of COVID-19, the best method for identifying the cause of death is still through a complete autopsy. Autopsies represent the ultimate diagnostic test, typically the gold standard, with 100% sensitivity and specificity for finding/excluding the causes of death [14].

The aim of this study is to describe gross and microscopic cerebrovascular pathology findings from the autopsies and correlate them with the clinical and laboratory characteristics of the COVID-19 patients. It is an extensive series of patients from the Brazilian Amazon with infection by SARS-CoV-2 and cerebrovascular disease, documented by complete autopsies, with extensive gross pathology documentation, and microscopic evidence of hemorrhages.

## Material and methods

This observational study ran between April 3^rd^ and July 24^th^, 2020, to describe the autopsy findings in hospitalized patients with suspected SARS-CoV-2 infection. All the tests and autopsies were performed at the Delphina Aziz Hospital, a tertiary hospital for the treatment of coronavirus patients in Manaus, Western Brazilian Amazon, and the largest public reference unit dedicated exclusively to the treatment of severe COVID-19 cases in the state, with an intensive care unit (ICU) capacity of 100 beds. At the beginning of the study, autochthonous SARS-CoV-2 transmission had already been recorded in Manaus, and the city became a major site of SARS-CoV-2 transmission in Brazil within a few weeks.

### Participants

Hospitalized patients were included if they had clinical and/or radiological suspicion of COVID-19. Suspicion of COVID-19 was defined by the presence or history of fever and any respiratory symptom, e.g., cough or dyspnea and/or ground-glass opacity or pulmonary consolidation observed on a computed tomography [CT] scan). Patients 18 years of age or older at the time of inclusion and either had SpO2 ≤ 94% with room air, or required supplementary oxygen, or required invasive mechanical ventilation were included. Children under 18 years of age were not included due to their known lower morbidity/mortality from COVID-19 [15]. Patients were enrolled before laboratory confirmation of COVID-19 to avoid treatment delays.

### Clinical and laboratory data

The hospital has all source documents registered online in an electronic medical recording system (Medview). Clinical analyses, laboratory examinations, and routine computed tomography scanning are also available on site.

According to the manufacturer’s recommendations, two nasopharyngeal swabs or one oropharyngeal swab (per institutional protocol) were used to extract viral RNA with the QIAamp Viral RNA mini kit. Subsequently, all swab specimens were tested for SARS-CoV-2 using the one-step multiplex RT-qPCR kit (Instituto de Biologia Molecular do Paraná, Curitiba, Brazil), following the manufacturer’s recommendations and targeting the virus nucleocapsid (N) (HEX) and ORF-1ab (FAM) genes and an endogenous human gene as the internal control (ROX). For all assays, specimens were considered positive if both viral targets, N1 and N2, showed cycle thresholds (CT) lower than 40.0.

### Autopsy and histologic examination

In all, forty-four patients were examined using the standard autopsy procedure. Autopsies were performed within 12 hours of death and, before the autopsy procedure, the body was embalmed with 10% formalin. After the opening of the cranial cavity, the brain and cerebellum were fixed with 20% formalin. According to adapted protocols, tissue sampling was performed systematically in six fragments, and 1–2 for gross lesions [16,17]. The fragments selected were fixed in 10% neutral buffered formalin, embedded in a paraffin block, 5-μm sections, and submitted to standard processing with hematoxylin and eosin staining. Special stains for *mycobacterium* bacilli or fungi were made when necessary (Wade or Grocott-Gomori, respectively). Evaluation of hematoxylin and eosin sections was performed by two pathologists who described the main pathologic findings, and agreement was by consensus.

### Statistical analysis

The patients were classified in two groups defined by the presence (1) or not (0) of hemorrhagic and/or thrombotic manifestations in the CNS. For descriptive analysis, variables with normal distribution were expressed as means and standard deviation, and those not normally distributed were expressed as median and interquartile ranges. Variables were compared between groups using the Mann-Whitney or T-tests when appropriate. To assess factors associated (Relative Risk) to nervous system hemorrhagic manifestations, variables were previously screened by simple log-binomial generalized linear regression, considering p<0.2 as a selection criterion for the multiple regression models, for which p<0.05 was considered as statistically significant. Stata^®^ software (version 13) was used for statistical analyses.

### Ethical considerations

This study was conducted in accordance with the principles of the Declaration of Helsinki and the Good Clinical Practice guidelines of the International Conference on Harmonization. The protocol was approved by the Brazilian Committee of Ethics in Human Research and was authorized by the National Research Ethics Committee accordingly (CAAE: 30152620.1.0000.0005). Signed informed consent was obtained from the legal representatives in all cases.

## Results

### Clinical and laboratory data

Of the forty-four patients that were autopsied, thirty-eight (86.4%) tested positive by RT-PCR for COVID-19, and six (13.6%) tested positive by the serological rapid test. Clinical and radiological findings were compatible with the infection. Macroscopic or microscopic hemorrhages were found in 23 of the 44 cases (52.3%). In this group, the mean age was 67, and most patients (n = 17; 73.9%) were male. Among the comorbidities in this group, arterial hypertension had the highest incidence (50%), followed by diabetes (54.55%) and obesity (23.81%) (Tables 1 and 2).

**Table 1:** Assessment of general data between study groups.

General Data	Presence Frequency (%)	Absence Frequency (%)	Relative Risk	95% CI	*p*
^#^Age	67 (20–84)	61 (35–83)	-	-	0.6541
^#^Hospitalization days	11 (02–31)	05 (01–36)	-	-	0.1192
^#^Body mass index	26.1 (21.5–34.6)	27.7 (25–34.1)	-	-	0.1948
^#^SpO^2^	95 (69–99)	96 (92–100)	-	-	0.2078
^#^PaO^2^/FiO^2^	163.3 (87.1–716)	163.3 (55–472.5)	-	-	0.9170
Gender (male)	17/23 (73.91)	15/21 (71.43)	1.063	0.5982 to 2.218	>0.9999
Race (brown)	16/23 (69.57)	18/21 (85.71)	0.6723	0.4071 to 1.281	0.2869
Invasive mechanical ventilation	22/23 (95.65)	16/21 (76.19)	3.474	0.9679 to 19.52	0.0883

#median (range)–Mann-Whitney test.

**Table 2:** Assessment of clinical data between study groups.

Clinical Data	Presence Frequency (%)	Absence Frequency (%)	Relative Risk	95% CI	*p*
**Comorbidities**					
Arterial hypertension	11/22 (50.00)	09/20 (45.00)	1.100	0.6094 to 1.983	0.7675
Cardiovascular disease	04/22 (18.18)	05/20 (25.00)	0.8148	0.3315 to 1.554	0.7139
Diabetes Mellitus	12/22 (54.55)	08/20 (40.00)	1.320	0.7375 to 2.416	0.3743
HIV	01/23 (4.35)	00/20	1.909	0.3878 to 2.547	>0.9999
Neurological disease	02/23 (8.70)	02/20 (10.00)	0.9286	0.2716 to 1.816	>0.9999
Obesity	05/21 (23.81)	05/17 (29.41)	0.875	0.3935 to 1.588	0.7268
Pulmonary disease	01/20 (5.00)	01/17 (5.88)	0.9211	0.1712 to 1.952	>0.9999
Renal chronic	03/22 (13.64)	01/16 (6.25)	1.342	0.5193 to 2.145	0.6245
**Hospitalization therapy**					
Antibiotic	22/23 (95.65)	19/21 (90.48)	1.61	0.6124 to 8.857	0.5988
Corticoid	08/23 (34.78)	03/21 (14.29)	1.6	0.8737 to 2.642	0.1685
Anticoagulant	21/23 (91.30)	17/21 (80.95)	1.658	0.7154 to 5.85	0.4029

Pulmonary disease: Asthma; Cardiovascular disease: Cardiac insufficiency and arrhythmia; Neurological disease: Ischemic stroke and Parkinson’s.

Among the patients in the group with hemorrhagic and/or thrombotic lesions in the CNS (HTM; hemorrhagic and thrombotic manifestations), most of the cases were hospitalized for 10 days before death. Although neurological manifestations of COVID-19 have been described, none of these were observed at the time of hospital admission. Corticosteroids, anticoagulants, and antibiotics were used to treat 34.78%, 91.3%, and 95.65% of the affected group during hospitalization. The initial gasometry revealed a critical PaO2/FiO2 ratio (median = 163.3) in both groups. The laboratory tests evidenced slightly higher levels of D-dimer (N = 6; median = 1237 g/L [983.5–3144]) in the affected group, whereas the lactate dehydrogenase (N = 4; Median = 829 u/L [619.5–3061]) was higher in the group with no hemorrhagic and/or thrombotic lesions. Both groups presented an increase in C-reactive protein and ferritin levels with a median of 92.3 and 1449, respectively, in the affected group, and 78.9 and 1495, respectively, in the other group. The nitrogen waste was elevated in both groups, and the creatinine level (median 1.81 mg/dL) was 30% higher in the group with hemorrhagic and/or thrombotic lesions, while the urea levels were similar in both groups, approximately 40% above the laboratory reference. Blood tests also revealed anemia, in which the moderate form (hemoglobin level: 10.2–13.4g/dL) was more prevalent in the patients with no hemorrhagic/thrombotic events (n = 13), while the severe form (hemoglobin level <8 g/dL) had the same prevalence in both groups (n = 2). Refer to Table 3 for further laboratory data.

**Table 3:** Laboratory tests and analysis of data between study groups.

Laboratorytests (Normal values in adults)	Presence	Absence	*p*
**Hematological data**	*Median (25 and 75 interquartile)*		
Hemoglobin (11.0–16.0g/dL)	11.4 (10.2; 13.4)	11 (10.2; 12.8)	0.5487
Platelet (100–300×10^3^/uL)	220 (183; 318)	249 (134.5; 363.5)	>0.9999
WBC (4–10×10^9^/L)	10.9 (7.65; 15.4)	12.6 (9.5; 17.32)	0.2089
Lymphocyte (1–4.8 x10^3^/uL)	5.6 (2.8; 10.1)	4.1 (2.3; 8.8)	0.3298
Neutrophil (% of WBC)	90.9 (85.9; 93.1)	90.9 (86.4; 93.8)	0.8115
**Biochemistryparameters**	*Median (25 and 75 interquartile)*		
D-dimer (up until 500ug/L)	1237 (983.5; 3144)	942.8 (414; 2811)	0.3543
DHL (240–480 U/L)	555 (402; 1073)	829.5 (619.5; 3061)	0.2571
INR (up until 1.00)	1.2 (1.09; 1.28)	1.2 (1.11; 1.31)	0.5569
C-reactive protein (0.3 to 10 mg/L)	92.3 (57.1; 211.2)	78.9 (67; 117.2)	0.2476
Ferritin (24 to 336 mg/L *men*)	1449 (576.3; 2885)	1495 (807.8; 3240)	0.9143
Glucose (45 to 96 mg/dL)	186.5 (146; 272.8)	159 (133.3; 291.8)	0.7394
Sodium (130–145 mmol/L)	141 (136.5; 143.5)	142.9 (138.8; 148)	0.1584
Potassium (3.6–5.0 mmol/L)	4.39 (4.2; 5.2)	4.34 (3.9; 4.6)	0.3231
Alanine aminotransferase (<36 U/L)	52.3 (43.8; 153)	41.8 (22.1; 75.6)	0.2496
Aspartate aminotransferase (<35 U/L)	44 (36.2; 78.3)	45.9 (38.4; 74)	0.9829
Direct Bilirubin (<0.3 mg/dL)	0.23 (0.22; 0.52)	0.43 (0.25; 0.78)	0.4240
Indirect bilirubin (0.2 to 1.2 mg/dL)	0.2 (0.08; 0.38)	0.31 (0.14; 0.48)	0.1717
Urea (7 to 20 mg/dL)	63.9 (38.4; 104)	62.1 (42.1; 139.2)	0.5490
Creatinine (0.6 to 1.2 mg/dL *men)*	1.81 (0.95; 3.6)	1.4 (0.92; 2.95)	0.5030
CK (55–170 U/L *men*)	136 (74; 267.1)	244.9 (81.3; 640.3)	0.6239
CKMB (0 to 4.9 ng/mL)	28.8 (15.6; 47.6)	46.8 (38.5; 107)	0.4000

The association analyses showed that the use of corticosteroid, anticoagulant and antibiotic had no statistical significance with a risk hemorrhagic manifestations in the nervous system. However, it is possible to note a statistical tendency that indicates that individuals with diabetes had a higher risk for the hemorrhagic manifestations (RR = 1.320, 95% CI = 0.7375 to 2.416, p = 0.3743) and this was not observed with the other comorbidities.

### Autopsy

**Fig 1:**
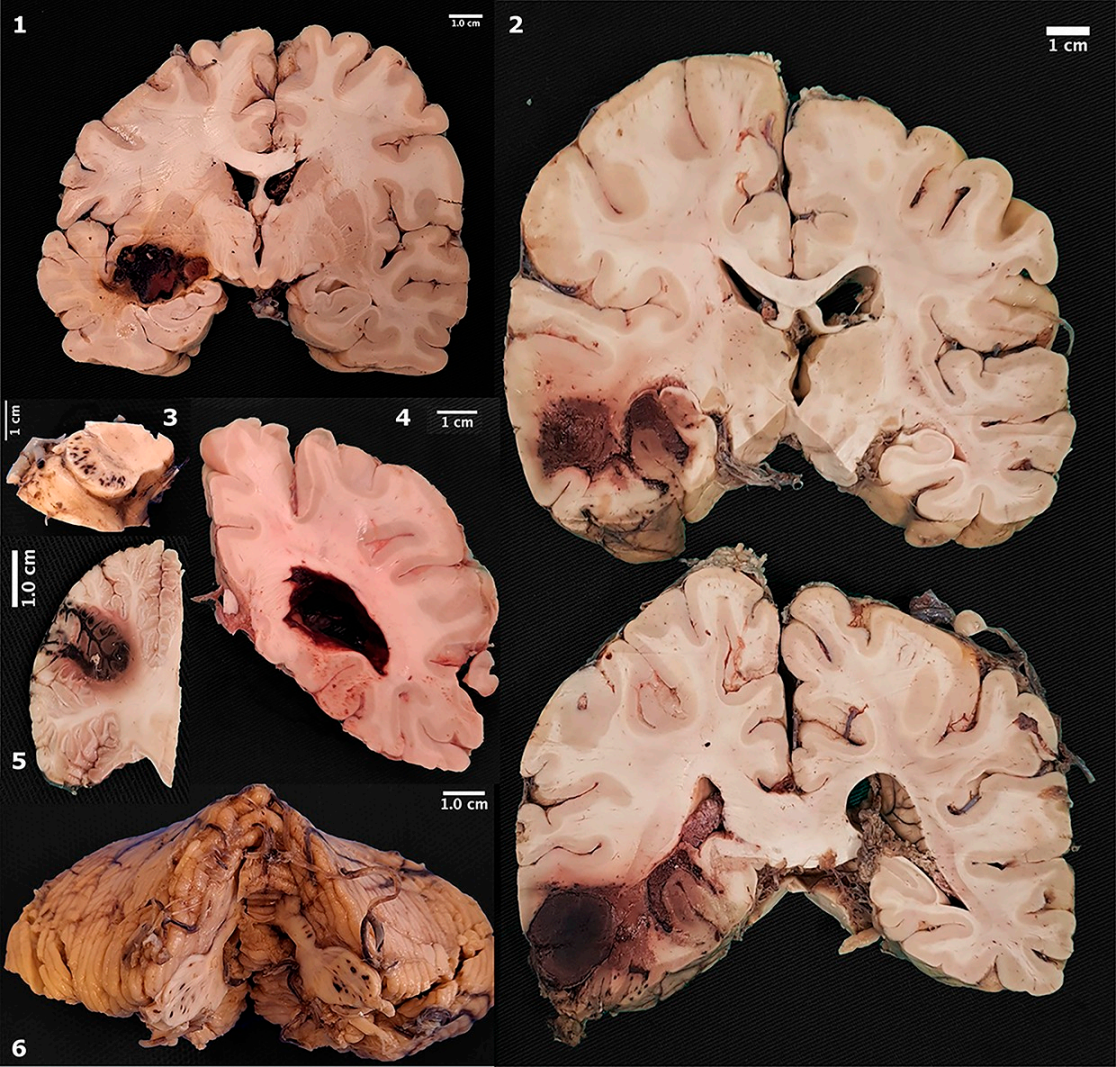
Gross pathology of macroscopic hemorrhages. 1. Hemorrhage in the putamen; 2. Extensive hemorrhage in temporal lobe; 3. Punctate hemorrhages in cerebral peduncles; 4. Lateral ventricle filled by blood; 5. Focal hemorrhage in cerebellar cortex; 6. Punctate hemorrhages in middle cerebellar peduncles.

**Fig 2:**
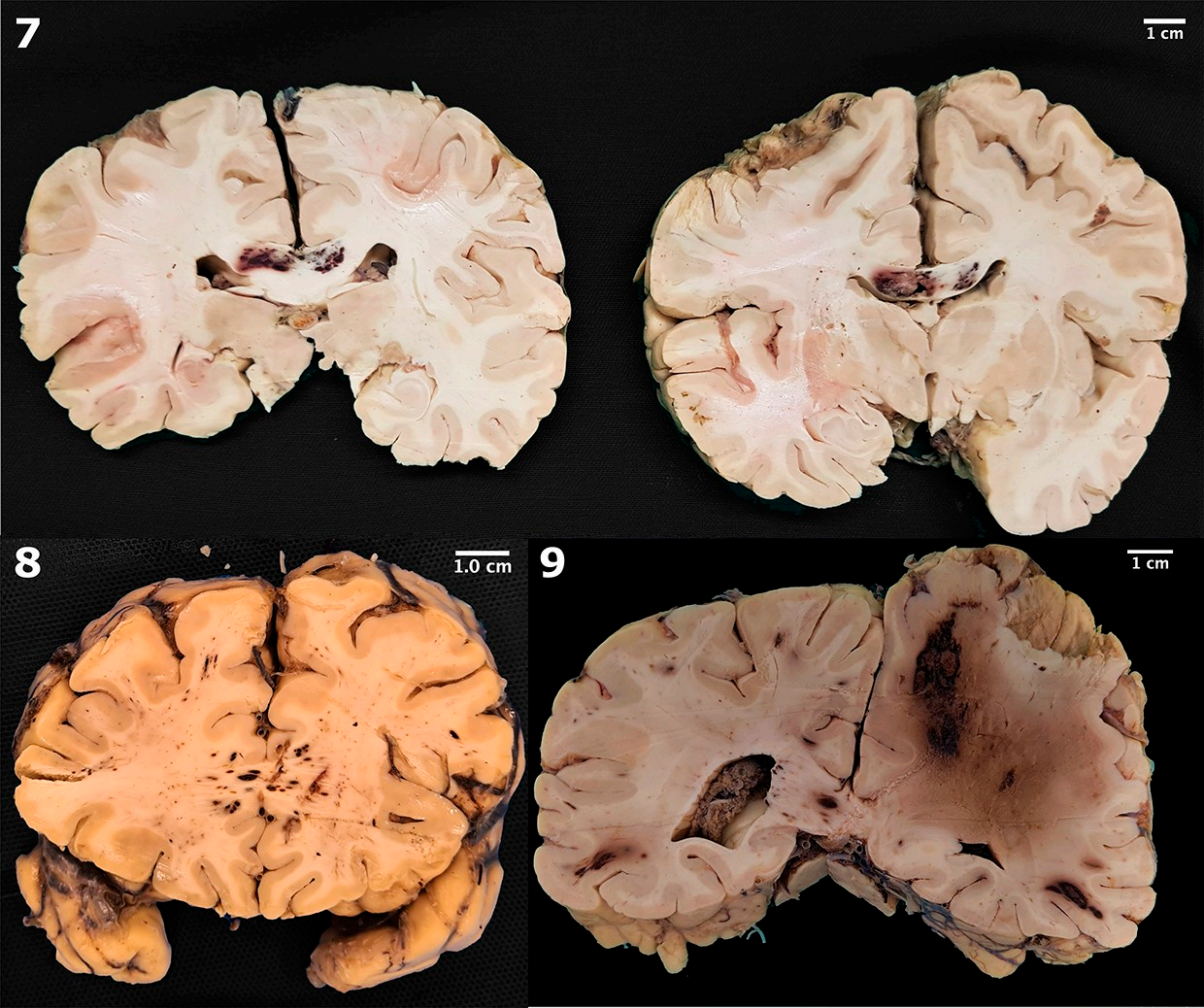
Gross pathology of macroscopic hemorrhages. 7. Hemorrhage in corpus callosum; 8. Hemorrhages in forceps major (occipitalis); 9. Extensive hemispheric hemorrhage, with intense deviation from the midline.

The gross postmortem examination of the brain revealed a large spectrum of hemorrhages, from spots to large and confluent areas in nine cases (20.4%) (Figs 1 and 2). The most common vascular territory identified affected the middle cerebral artery (three cases). Other cases affected were (one case, each): anterior cerebral, anterior inferior cerebellar, striatum, and basilar arteries (Table 4).

**Table 4:** Distribution of macroscopic hemorrhages and vascular impairment.

Topography of hemorrhage	Vascular territory	Laterality	Pattern
Hemorrhage in corpus callosum	Anterior cerebral	Bilateral	Focal
Focal hemorrhage in cerebellar cortex	Anterior inferior cerebelar	Unilateral	Focal
Extensive hemorrhage in temporal lobe	Middle cerebral	Unilateral	Focal
Hemoventriculum	Intraventricular hemorrhage	Bilateral	Diffuse
Extensive hemorrhage from temporal lobe to the occipital lobe	Middle cerebral	Unilateral	Diffuse
Hemorrhage in temporal lobe, putamen, and globus pallidus. Lateral ventricle filled with blood	Middle cerebral Lenticulostriate arteries	Unilateral	Focal
Large hemorrhage in claustrum, putamen e globus pallidus. Punctate hemorrhages in cerebral peduncles and middle cerebellar peduncles	Lenticulostriate arteries Basilar Posterior cerebral Anterior inferior cerebelar	Bilateral	Diffuse
Petechial hemorrhage in pons	Basilar	Unilateral	Focal
Lateral ventricle filled with blood	-	Bilateral	Diffuse

The histological examination of these lesions revealed erythrocytes in perivascular space and/or microhemorrhages (21; 47.7%) and thrombi in small-caliber vessels (3; 6.8%). Intraventricular hemorrhage was observed in three cases (6.8%).

**Fig 3:**
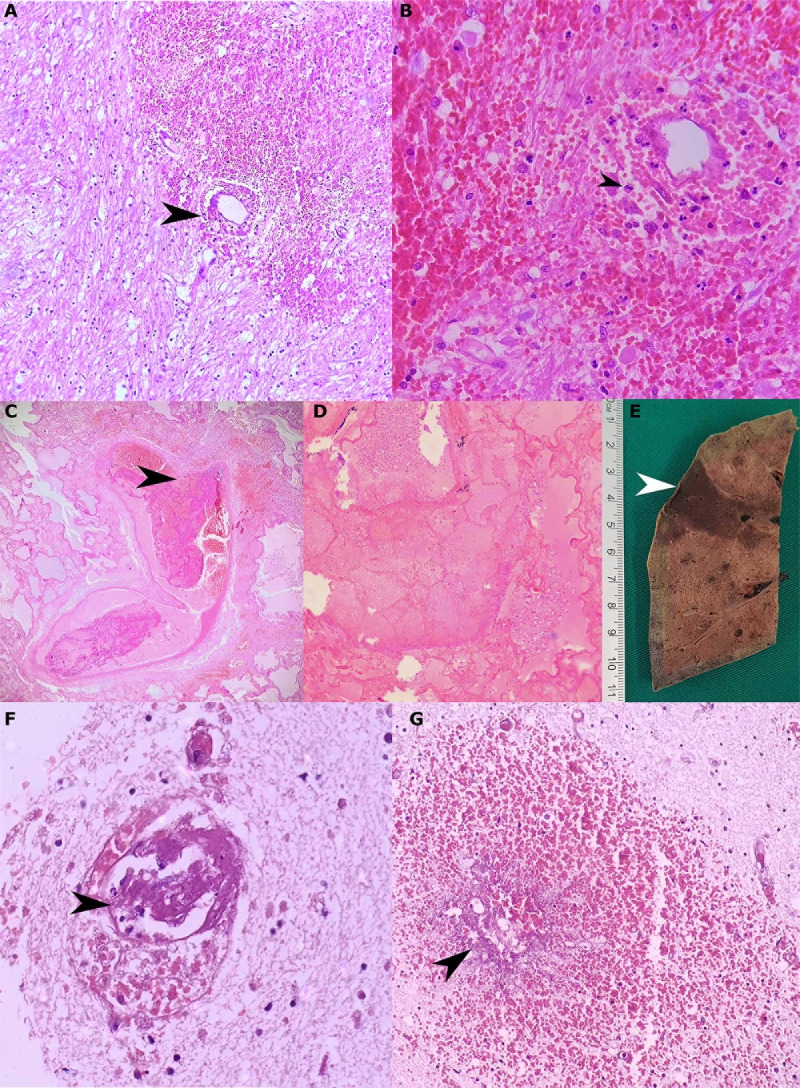
Microscopic thrombo-hemorrhagic alterations and systemic complications. Intraparenchymal hemorrhage due to rupture of the vascular wall (black arrowhead, A). Intraparenchymal hemorrhage with discrete inflammatory infiltrates of neutrophils (black arrowhead), lymphocytes and histiocytes (B). Thrombi in a large vessel (C), causing pulmonary infarct (D and E), with microthrombi in cerebral parenchyma (black arrowhead, F). Petechial hemorrhage with wall vessel necrosis (G). (hematoxylin and eosin. A: 100x, B: 200x, C: 100x, D: 200x, F: 400x, G: 200x).

**Fig 4:**
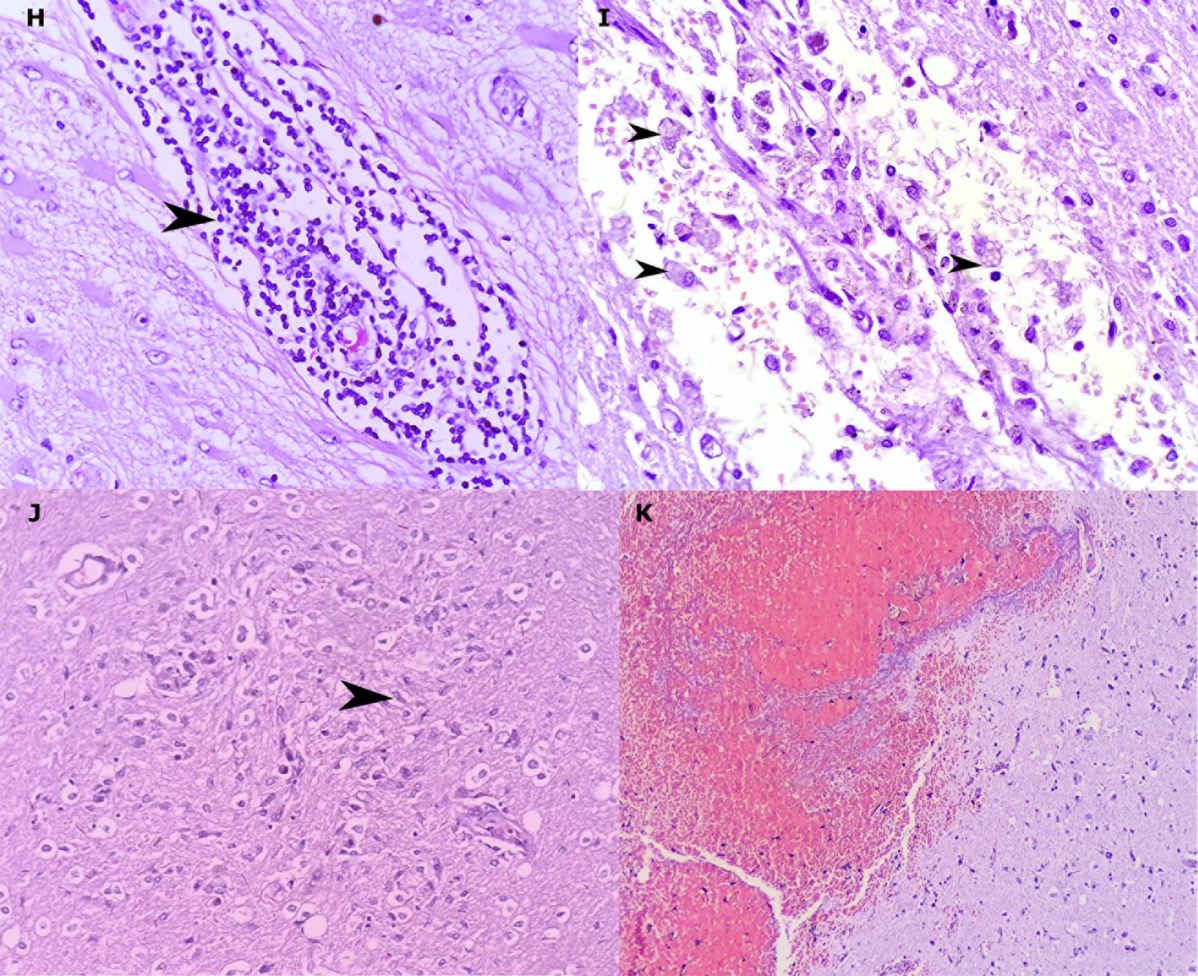
Inflammatory cells and reaction processes in the examined brains. Lymphocytes (arrowhead) forming bulky perivascular cuffing (H). Macrophages (arrowhead), red cell diapedesis and edema around blood vessels (I). Microglial nodule (microglia cell, arrowhead) (J). Occurrence of intraparenchymal hemorrhagic phenomenon without association with inflammatory process (K). (hematoxylin and eosin. H: 400x, I: 200x, K: 200x).

In some cases, we observed mild brain swelling and hemorrhage, which was secondary to vascular damage. In one case, the vascular thrombotic disease prompted a systemic impairment, affected the pulmonary vascular bed and caused tissue infarction. Microthrombi affected the cerebral vessel and macrophages were present at the periphery of the lesions of the genu of the corpus callosum (Figs 3 and 4). The cut-off considered for perivascular cuffing was 12 lymphocytes, but up to 43 lymphocytes were observed around the vessels in 17 cases (38.6%) (Table 5).

**Table 5:** Hemorrhagic and thrombotic manifestations: Patterns and distributions.

	Frequency (%)
*Patterns*	
Macroscopic alterations	9 (39.1)
Focal	5 (55.5)
Diffuse	4 (44.4)
Unilateral	5 (55.5)
Bilateral	4 (44.4)
Hemorrhagic infarct	1 (11.1)
Only microscopic alterations	14 (60.9)
Acute (predominance of erythrocytes)	23 (100)
*Macroscopic topography of hemorrhages* (n = 9)	
White matter	5 (55.5)
Gray matter	3 (33.3)
Basal ganglia	2 (22.2)
Brainstem	1 (11.1)
Cerebellum	1 (11.1)
Subarachnoid	1 (11.1)
Intraventricular	3 (33.3)
*Microscopic alterations* (included all cases evaluated—n = 44)	
Congestion and/or edema	23 (52.2)
Erythrocytes in perivascular space and/or microhemorrhages	21 (47.7)
Lymphocyte cuffing	17 (38.6)
Demyelination foci	5 (11.4)
Microglial nodules	5 (11.4)
Thrombi in small vessels	3 (6.8)
Hemorrhagic infarct	3 (6.8)
Ischemic infarct	2 (4.5)
Vasculitis	2 (4.5)
Subarachnoid hemorrhage	1 (2.2)

## Discussion

In the severe acute respiratory syndrome described in March 2003 (SARS-CoV-1), thrombotic events were identified in hospitalized patients [18]. Neurological manifestations caused by SARS-CoV-2 have affected 36.7% of patients [7]. This autopsy study showed a frequency of 9/44 (20.4%) macroscopic and 14/44 (31.8%) microscopic hemorrhages or thrombotic phenomena in the nervous system. Tang et al. [19] suggest that hemostatic abnormalities, such as high D-dimer and fibrin degradation product (FDP) levels, longer prothrombin time, and activated partial thromboplastin time (aPTT), affect COVID-19 patients and are more frequent in severe disease when compared to survivors on admission [19]. Similarly, our patients with hemorrhages presented a trend for higher aPTT and D-dimer.

In COVID-19, some factors in thrombotic or hemorrhagic events should be discussed. Several drugs have been investigated for treatment of severe SARS CoV-2, but these medications may have adverse interactions with antiplatelet agents and anticoagulants. Some agents, such as bevacizumab and fingolimod, have been associated with excess or reduced risk of thrombotic events or thrombocytopenia in prior studies of non-COVID-19 populations [20]. In our study, the association between the use of corticoids, anticoagulants, or antibiotics with hemorrhagic/thrombotic events was not statistically significant.

Neurological manifestations, such as acute cerebrovascular diseases, impaired consciousness, and skeletal muscle injury, were more common in severe COVID-19 and presented a frequency of 36.4% in hospitalized patients [7,21]. Clinical and radiologic data on neurological conditions were limited in our study because of disease severity since patients required orotracheal intubation and sedation. Comparing the groups in our case series, these patients presenting central hemorrhagic lesions were older (mean of 67 years of age) than the non-affected group (mean of 61 years of age), which was also observed by Ling Mao et al. [7] in patients with neurological symptoms and COVID-19 in Wuhan, China. In other studies, the neurological manifestations were due to subarachnoid hemorrhage [9], ischemic strokes [22–24], acute hemorrhagic necrotizing encephalopathy [11], vascular and acute disseminated encephalomyelitis‑like pathology [25], encephalitis, and meningitis [8,26]. In our autopsies, we described macroscopic and microscopic hemorrhages, and we focused only on these manifestations, without exploring encephalitis, meningitis, or inflammatory alterations, since these will be reported in another study.

A pronounced CNS involvement with pan-encephalitis, meningitis, and brainstem neuronal cell damage in six autopsied patients was described by Weyhern et al. (2020) in patients from Germany. Petechial bleeding was found in four patients (8.8%), and only two of them were younger than 60 years old. In our patients, erythrocytes in perivascular space and/or microhemorrhage were found in 21/44 (47.7%). In a small series of COVID-19 patients younger than 50 years old, the vascular territory most affected was the left middle cerebral artery (3/5 patients), and three patients presented elevated levels of D-dimer (up to 500 ng per milliliter) [27]. In a study of 14 COVID-19 patients conducted by Bradley et al. (2020), only five patients were submitted to a brain examination, and one showed neuropathological alterations, with a scattered punctate subarachnoid hemorrhage and a rare microhemorrhage in the brainstem [28]. In other autopsy studies, samples from the brain are limited [29] or unavailable for gross pathology or histopathological evaluation [30]. In the postmortem case series of Matschke et al. (2020), which involved 43 autopsies, there was no evidence of cerebral bleeding or small vessel thrombosis [31]. In our patients, we frequently found microscopic petechial hemorrhages, similar to what is described in cerebral malaria [32]. The absence of large autopsy series describing the neuropathology associated with COVID-19 is notable, and the small amount of published data is also contradictory; therefore, any published data must be carefully reviewed so as not to increase the current disparity [33].

Intracranial hemorrhage can occur spontaneously in hypertensive or atherosclerotic patients and is secondary to the rupture of a vessel (due to high blood pressure or secondary to a spasmodic softening), rupture of capillaries or venules (due to fluctuating blood pressure or disorders of venous circulation), and due to diapedesis of red blood cells. In a series of cases described by Mutlu, Berry, and Alpers (1963), massive cerebral hemorrhage was secondary to diapedesis in 40% of cases, wherein rupture of capillaries or venules results in an effusion of erythrocytes. Due to the lack of clinical, radiological, and angiographic data, the etiology of the bleeding could not be determined in our patients. There are no extensive studies in the literature about the etiology of intracerebral hemorrhage in patients with COVID-19.

In our patients, there were no unequivocal signs that were suggestive of aneurysmal ruptures, or vascular malformations, which are findings that are consistent with hypertensive cerebrovascular disease or other macro or microscopic changes that indicate the significant etiology of hemorrhagic manifestations. However, current data on the pathophysiology of COVID-19, especially on damage to the central nervous system, cannot, with certainty, establish a causal relationship between a viral infection and hemorrhagic manifestations. Despite the absence of data, endotheliosis [34], larger vessel strokes [27], thrombotic and hemorrhagic complications have been described in COVID-19 [35].

The elevation of D-dimer levels, also observed in other case series [22,36,37], is directly related to the severity of the disease and can be used as one of the indicators of COVID-19 progression [7]. High levels of D-dimer suggest hypercoagulability, but do not establish the causality between cardiovascular disease (CVD) and COVID-19 [24].

Because of their potent anti-inflammatory properties, corticosteroids have been used for decades to treat many diseases. However, the literature has shown that the use of corticosteroids can cause lower coagulation factors [38–40]. It is known that glucocorticoid may induce hyperglycemia and has significant clinical implications in patients with and without diabetes mellitus [41,42]. The toxicity of hyperglycemia in critically ill patients can be explained by cellular glucose overload. This imbalance leads to excessive glycolysis and oxidative phosphorylation and increases reactive oxygen species (ROS) production. The ROS excess surpasses and/or compromises the cellular detoxification pathway and ultimately induces apoptosis [43].

The use of corticosteroids has also been associated with an increased risk of venous thromboembolism [44,45]. Majoor et al. (2016) investigated whether a 10-day prednisolone burst therapy would activate hemostasis in healthy individuals [46]. The authors concluded that oral prednisolone induces a procoagulant state, suggesting that corticosteroid treatment may increase the thromboembolic risk in patients with inflammatory diseases. The evidence presented by the authors on the greater risk associated with the repositioning of the medication in question is notorious (3.06; 2.77–3.38). A severely low platelet count is also an important manifestation of critical SARS-CoV-2 infection, as well as an independent risk factor for acute cerebrovascular events. The ACE2 protein has been identified as a functional receptor for SARS-CoV-2, and is expressed in several human organs and tissues, such as endothelial cells [47]. ACE2 performs an important role in, but is not limited to, the cardiovascular and renal functions, including blood pressure regulation. Once SARS-CoV-2 reaches the circulation and binds to the ACE2 receptor, it may increase blood pressure, and augment the risk of intracranial hemorrhage, especially in previously hypertensive patients [48]. Platelets are considered critical mediators of inflammatory processes and can be indicators of infectious agents. Activation and interactions among them and macrophages, monocytes, endothelial cells and lymphocytes play a critical role in the procoagulant effect [49]. A study of 191 patients with COVID-19 suggested coagulopathy as a potential life-threatening factor since 50% of those who died had some clotting disorder compared to 7% of survivors [50]. Despite that, in our cohort, only one patient had severe thrombocytopenia.

The limitations of our study include the absence of radiological data and clinical signs and symptoms, which was due to the sedation and the very severe clinical condition of the affected patients. We cannot safely establish the relationship between hemorrhagic manifestations and infection with the new coronavirus; however, autopsies in patients in this study reveal, in loco, the severity and potential lethality of these manifestations.

Autopsies in patients with COVID-19 help to elucidate the natural history of the disease and may aid the development of ideal therapeutic schemes when correlated with the clinical features of infected individuals. They can lead to better clinical practice by helping to understand the etiopathogenic mechanisms of little-known diseases and elucidating impairments hitherto not considered for evaluation by health care teams. Macroscopic and microscopic findings, which are essential for understanding the mechanism of death in patients, can also reveal other undiagnosed clinical conditions *in vivo*. In our study, complete autopsies provided valuable information about the neuropathological parainfectious processes in COVID-19, such as hemorrhagic damage. Thus, further studies are needed to define clinical and radiological monitoring protocols and strategic interventions for patients at risk of adverse and fatal events, such as the extensive hemorrhage described here. It is imperative that doctors are aware of the comorbidities and medications used to treat patients with COVID-19 to prevent CNS hemorrhagic and thrombotic events.

## Supporting information

S1 File (XLSX)

S2 File (XLSX)
