# Expression of Concern: Hemorrhagic and thrombotic manifestations in the central nervous system in COVID-19: A large observational study in the Brazilian Amazon with a complete autopsy series

**DOI:** 10.1371/journal.pone.0348044

**Published:** 2026-04-28

**Authors:** The PLOS One Editors

## Notice of Republication

The article was republished on April 13, 2026, to replace S2 File with a corrected version.

Following publication of this article [1], concerns were raised about incomplete reporting of the study design and omission of relevant patient treatment information.

Upon editorial follow-up, corresponding author RAdAP confirmed that the patients whose autopsies are described in the article were participants in the Clorocovid-19 trial [2] (registered with Clinicaltrials.gov as NCT 04323527), in which patients were randomized to receive either high- or low-dose chloroquine diphosphate with the trial later evolving to compare low dose with placebo. Based on information received on follow-up, the *PLOS One* Editors are satisfied that the study reported in [1] was covered by the ethical approval CAAE: 30152620.1.0000.0005 issued by Comissão Nacional de Ética em Pesquisa (CONEP) for the Clorocovid-19 trial.

The autopsy series [1] investigating central nervous system (CNS) hemorrhagic and thrombotic events in COVID-19 reports association analyses with corticosteroid, anticoagulant, and antibiotic treatments, but did not report that patients in the study received chloroquine diphosphate treatment as clinical trial participants. PLOS consulted a member of the *PLOS One* Editorial Board who advised that chloroquine diphosphate treatment should have been reported and its clinical implications discussed, with appropriate association analyses carried out and the limitations of those analyses discussed.

Corresponding author RAdAP indicated that they did not consider it necessary to report chloroquine diphosphate treatment because in their view there is no rationale to believe that chloroquine could have changed the CNS macroscopic or microscopic lesions, or the interpretation of the findings from the series of autopsies. They noted that no neurological findings were reported in previous case reports of patients treated with high dose chloroquine [3]. They also noted that exposure to the drug in the clinical trial [2] was heterogeneous, and the sample size for [1] was not powered for comparisons based on chloroquine diphosphate treatment status.

RAdAP provided an updated S2 File which includes individual-level information about chloroquine diphosphate treatment. They noted that four subjects were allocated to the high-dose arm of the original study; however, none of them actually completed the entire regimen. They stated that they did not observe pathological findings specific to these four cases.

Given the sample size limitation and in the absence of further analyses, the *PLOS One* Editors note that we cannot ascertain whether any of the findings reported in the article may be associated with chloroquine diphosphate treatment status. The *PLOS One* Editors issue this Expression of Concern to inform readers of the above issues.
